# On the Treatment of Eczema

**Published:** 1864-04

**Authors:** J. Wallace

**Affiliations:** Dalston, Carlisle


### ON THE TREATMENT OF ECZEMA.

By Dr. J. WALLACE, Dalston, Carlisle,

[We give one out of several cases related by Dr. Wallace, to illustrate a mode of treating eczema, which we have ourselves followed for many years, and which we know is usually most successful. The plan of treatment consists in getting the patient or nurse to keep the parts continually moist with lint saturated with a very weak alkaline lotion. The proper strength is about half a drachm of carbonate of soda to eight ounces of water. It is a *sine qua non* that the lint should never be allowed to dry, otherwise the application is worse than useless.]

*Case.*—A. B., a quarryman, aged thirty-four, consulted me in October, 1861, who had a skin disease of six years' duration, extending over both cheeks, of a circinate form, and communicating over the nose. Had been in two infirmaries, consulted many medical men, and had all sorts of ointments and lotions applied, which invariably made the disease no better, and oftener worse. In every other respect he was healthy. On examination, the diseased surface presented the appearance of dry, indurated, laminated scales, firmly impacted, overlaying and dovetailing with each other, so as to form a crust about one-eighth of an inch, nearly, in thickness, over both cheeks. On the nose it was thinner and moist. Round the margins was a slight erythematous blush, the incrusted morbid epithelium flattening off to the healthy skin, and at the junction of the two a watery fluid, he said, sometimes oozed. On careful and repeated inquiry, it was found that at first there was no hard incrustation, but that a raw surface had oozed a watery fluid, etc.; in short, that it was eczema utterly changed in its character by the multiplicity of intended remedial applications—the last one having been mercurial ointment. He was ordered to apply the alkaline wash—a drachm of carbonate of soda to half a pint of pure water—by means of lint and a mask; the lint to be moistened three or four times daily with the lotion, and, as he was working in a quarry, to be moistened with pure water frequently from without, as he found it becoming dry. Also to take fifteen minims of the tincture of the muriate of iron, thrice daily, in water. In four days the crusts came off, and a healthy skin was partly formed over the diseased surface. It went on improving, and in three weeks the disease had vanished, and his face was, as he expressed it, "like other people's."

I put more faith in the external application than the internal alkaline remedies with arsenic, although I do not ignore the latter; but I have cured eczema, again and again, by means of the former only. What the therapeutic action of the alkaline lotion may be, is a matter of speculation. It has peculiar local sedative effects, while it is also gently stimulating; but may there not be some peculiar organic acid which by an irritating influence raises the eczematous vesicles and sustains the acrid discharge, which it neutralizes and ultimately destroys, so as to permit the speedy formation of new cuticle? Whether or not this may be the case, pathology will ultimately reveal; but, in corroboration of this idea, I have often found the alkaline lotion of great use in old constitutional and irritable ulcers, evidently from the same cause; and, indeed, the chief ingredient in every old wife's herb-salve in the country is alkaline.—*Lancet, Aug.* 29, 1863, *p.* 259.

